# Chronic mass psychogenic illness among women in Derashe Woreda, Segen Area People Zone, southern Ethiopia: a community based cross-sectional study

**DOI:** 10.1186/s13033-018-0207-1

**Published:** 2018-06-07

**Authors:** Moges Ayehu, Misganu Endriyas, Emebet Mekonnen, Mekonen Shiferaw, Tebeje Misganaw

**Affiliations:** 1 College of Medicine and Health Sciences, Hawassa University, Hawassa, Ethiopia; 2 SNNPR Health Bureau, Hawassa, Ethiopia

**Keywords:** Mass psychogenic illness, Mass hysteria, Epidemic hysteria, Derashe Woreda, Breast cancer, SNNPR

## Abstract

**Background:**

Outbreaks of mass psychogenic illness (MPI), which are a constellation of physical signs and symptoms suggestive of organic illness with no identifiable causes. MPI has been documented in numerous cultural, ethnic, and religious groups throughout the world. The aims of this study were to document the nature and impacts of the illness, to assess interventions, and to come up with recommendations and management formulations for dealing with such kinds of outbreaks in the future.

**Methods:**

Community based cross-sectional study was conducted in June, 2015 in Derashe Woreda, Segen Area People Zone of the Southern Nations Nationalities and People’s Region. Women with complaints of breast cancer but with no objective findings were the subjects of the study. Ninety-seven women were investigated using a semi-structured questionnaire for quantitative study. Two focus group discussions with seven affected and seven non-affected women and four key informant interviews were conducted using guiding questionnaires. Quantitative data was analyzed using SPSS version 20 software packages while qualitative data was analyzed manually going through thematic areas.

**Result:**

The ages of the ninety-seven study participants ranged from 17 to 56 years, with a mean (SD) of 32.8 (8.7) years. Onset of illness was dated back to the year 2012 following the death of a 43 year old socially active woman with complications of breast cancer. Following her death many women started to report multiple vague physical complaints similar to those of the deceased woman. Even though the responses from the study participants did not specifically point to a single possible cause and means of transmission, high numbers of women believed the source of their illness could be punishment from God while some said that the cause of their suffering could be environmental pollution. Since the illness was taken to be contagious, affected women faced stigma and discrimination. Moreover, school activities and social gatherings were limited significantly.

**Conclusion:**

Unrealistic and exaggerated rumors and inadequate explanations about the nature and spread of the illness were the main contributing factors for the spread and prolongation of the outbreak. An organized intervention, clear and adequate explanations about the nature and transmission of the illness can contain MPI within a short period of time.

## Background

Mass psychogenic illness (MPI) or mass hysteria has been defined as a group of physical signs and symptoms which suggest the presence of organic illness but with no any clinical and laboratory evidence of disease, and as disease that affects more than one person who share a conviction of having a similar illness [1]. It is also defined as “the rapid spread of illness signs and symptoms affecting members of a cohesive group, originating from a nervous system disturbance involving excitation, loss or alteration of function, whereby physical complaints that are exhibited unconsciously have no corresponding organic aetiology” [2].

The outbreak usually follows an environmental trigger or illness in an index case and spreads rapidly by audiovisual cues, and is often aggravated by an exaggerated emergency or media response [3–6]. It commonly affects people who live in groups such as nunneries, boarding- houses, schools, prisons and religious institutions [7, 8]. Various studies in the past have shown that 50% of reported outbreaks of MPI cases came from schools, followed by factories (29%), villages (10%) and families and other institutions (9%) [5, 7, 9].

MPI can be manifested in two forms: “mass anxiety hysteria” and “mass motor hysteria”. The former consists of episodes of acute anxiety, occurring mainly in school children. Prior tension is absent and the rapid spread is mainly due to visual contact with a person affected by the illness”, and many of the victims recover within 24 h. But the latter case consists of abnormalities in motor behavior, prior tension is usually present and any age group can be affected by it. Moreover, index cases can be identified, the spread is gradual and outbreaks may be prolonged from weeks to months [10].

Symptoms of MPI reported in more than one-third of individuals include headache, dizziness or light-headedness, nausea, abdominal cramps or pain, cough, fatigue, drowsiness or weakness, sore or burning throat; whereas hyperventilation or difficulty of breathing was reported in one-fifth of individuals [11]. These symptoms together with other symptoms such as shaking, twitching, trouble walking, fainting, vomiting, palpitation, anxiety, itching, watery eyes, chest pain, communication difficulties, uncontrollable laughing, and trance states have also been commonly reported in African settings [5, 12–15].

In Africa evil spirit, witchcraft, Satanism or failure to perform cultural and religious rituals as needed, have been mentioned as the causes of their illnesses [5, 12, 13, 16]. When it comes to western setting, however, the effects of toxic chemicals and environmental pollution have been replacing previous beliefs similar to those in Africa [6]. Because of these attribution styles, in Africa, many victims of MPI commonly seek treatment from religious and traditional healing sites [5, 12, 13]. Though there is no conclusive evidence about the causes of MPI, the following are postulated causes: psychological factors, environmental factors, different stressors, conflicts, lower level of education, lower socioeconomic status, minority race, and history of abuse or trauma [17–19].

Since the year 1374, outbreaks of mass hysteria have been documented in various cultural [20–22], ethnic [23–25], and religious [25–27] groups throughout the world. Such outbreaks were also reported in different African settings including Ethiopia [12, 14, 28–31]. Despite the difference in culture, religion and age range, the clinical presentation of the victims of epidemic hysteria are somehow similar.

In all of these incidents, the illness was mainly attributed to “evil–devil force”; management was provided in fragmented ways and it brought intense public terror to the extent of compromising school and occupational activities. Though there is an increase in awareness among health professionals, mass psychogenic illness is still underappreciated, under reported and still causing significant health and social problems in our country.

Since the beginning of 2012, following the death of a 43 year old socially recognized woman, several women were reporting of having a breast cancer in Derashe Woreda (a district administrative structure of about 100,000 peoples), Segen Area People’s Zone of SNNPR. The index patient died of metastatic breast cancer, a few months after having mastectomy done on one of her breast. Following her death the number of women who reported similar complaints increased to 120 within 3 years. Among these women, half of them had reported the complaints to nearby health institutions a year before this study was carried out. Fragmented medical treatments and psychoeducation services were provided here and there by nearby health institutions. However, the interventions and explanations forwarded by health professionals could not satisfy the victims and their families. As a result, the victims started visiting traditional and religious healing sites as treatment options. The traditional healers used hot metallic rode to cauterize the affected area as a treatment. In addition, these traditional healers also advised the affected individuals to apply herbs and to drink herbal juices which they provided. Later, when the issue got a huge public attention, the Regional Health Bureau deployed an emergency team consisting of public health officers, general practitioners, gynecologists and different other stakeholders to the area.

The team examined 92 women who reported of having breast cancer. Among these women 87 (94.6%) of them did not have any objective finding which suggested the presence of breast cancer except breast scars which were noted in some of them. For the remaining five women, bilateral breast lump in 2 (2.2%), axillary lymphadenopathy in 2 (2.2%) and breast ulcer (following cauterization as traditional treatment) in 1 (1.1%) were found. Finally, the team concluded that there was no breast cancer but there could be psychological explanation for the women’s complaints. So, this study was designed to document the nature and impact of the illness, to assess the interventions and come up with recommendations and management formulations for dealing with such kinds of outbreaks in the future.

## Methods

A community based cross-sectional study was conducted in Derashe Woreda, Segen Area People’s Zone of SNNPR, in June 2015. Woreda is a district administrative structure of about 100,000 peoples. Derashe has ten kebeles (local administrative unit or sub-unit within the Woreda) with different ethnic groups. Among these ethnic groups, Derashe, Gawwada, Mossiya, Kusumie, Mashole, Konso, Gamo and Amhara comprise the majority. Only the two Kebeles (Bussa Bassa and Bussa Killa) were affected by the outbreak.

For quantitative data, ninety-seven women with complaints of breast cancer, but without objective physical and laboratory findings were included. Two focus group discussions (FGDs) (seven women from affected groups and another seven from non-affected groups) and four key informant interviews (KIIs) were conducted as part of the qualitative study. A semi-structured questionnaire containing the socio-demographic variables, symptom complex, onset and duration of illness for quantitative data gathering was prepared by the investigators. In addition, participants were asked about illness attribution, information source, presence of recent psychosocial stress, their perceptions towards measure taken by officials, presence of similar illness in the past, and presence of substance use or mental illness at present or in the past. Focus group discussions and key informant interviews were facilitated by using a guiding questionnaire developed by the investigators. Home to home interviews with affected patients were conducted using line list of patients and local guides from the community. Participants were interviewed alone to secure confidentiality and to avoid information bias. Formal physical assessments were done at the end of each interview in order to rule out recent underlying medical illnesses.

Quantitative data was collected using four BSc degree holder public health officers who can speak the local language while qualitative data was gathered by two investigators. In addition, one supervisor was assigned to organize, facilitate, and control the whole process of the data collection. Filled questionnaires were checked for completeness and consistency of the information on daily basis. Quantitative data was entered and analyzed using SPSS for windows version 20.0. FGDs and KIIs were facilitated by using guiding questionnaire developed by the investigators. The developed guides were reviewed by research team and program experts for reducing ambiguity, leading questions, emotive questions and stressful questions; thereby to address trustworthiness. Guiding questionnaires were prepared in English and translated into local language (Amharic) by investigators. Two investigators at a time (one interviewer, one note taker) conducted interviews and discussions.

Verbatim transcription of FGDs and KIIs was done, and transcriptions were translated to English. We used framework analysis as it is tailored for policy research and allows for inclusion of a priori and emerging concepts [32–34]. Even though framework analysis is inductive in its approach, studies suggest that it can be used with deductive [34] and descriptive [35] approaches. So, we used deductive and descriptive approaches to summarize data. Transcriptions were read multiple times to validate transcription and familiarize with content. Preliminary themes were prepared by categorizing data into groupings. Key phrases and quotes were coded, classified and categorized repeatedly for deeper understanding. Preliminary themes were refined subsequently and finally, groups of related data were clustered to similar themes. Quotes that best described main themes were chosen and presented. Though we noticed response overlap in the majority of the qualitative and quantitative data finding, we have not done triangulation in the analysis process; so this should be taken into consideration when you read the finding of this study.

Ethical clearance was obtained from the Ethical Review Committee of Regional Health Bureau. All participants gave verbal consent to participate after through explanation of benefits of study and all the information obtained was anonymous and kept confidential.

## Results

### Socio-demographic characteristics of the participant

For quantitative investigation, a total of 97 women were assessed. The ages of the respondents ranged from 17 to 56 years, with a mean (SD) of 32.8 (8.7) years. Sixty-nine (71.1%) of them were from Bussa Bassa Kebele while the rest were from Bussa Killa Kebele. Ninety-two (94.8%) of the participants were followers of protestant religion. Eighty-eight (90.7%) of the study participants were married. Moreover, seventy-five (77.3%) of the participants were house wives by occupation. More than three-fourth of participants did not attend formal education from which 76.3% were unable to read and write while 4.1% were able to read and write from the skills they got from informal educational setting like church, family member, and friends. Except for one participant, the rest were from Mossiya ethnicity (see Table 1).

**Table 1:** Socio-demographic characteristics of the study participants

Characteristics categories	Number	Percent
Age		
17–30	42	43.3
31–40	41	42.3
41–56	14	14.4
Religion		
Protestant	92	94.8
Orthodox	3	3.1
Traditional religion	2	2.1
Got		
Dubayisho	30	30.9
Mender	28	28.9
Makiyo	23	23.7
Hoda	3	3.1
Kereza	4	4.1
Lemo	3	3.1
Osa	3	3.1
Olsaka	3	3.1
Marital status		
Married	88	90.7
Single	6	6.2
Widow or divorced	3	3.1
No of children		
1–3	29	29.9
4–6	45	46.4
7–10	17	17.5
Occupation		
House wife	75	77.3
Farmer	18	18.6
Others	4	4.1
Educational status		
Unable to read and write	74	76.3
Able to read and write	4	4.1
1–6	14	14.4
7–12	5	5.2

### Symptom profile

In addition to the complaints of having breast cancer, a majority of the study participants were complaining about the following symptoms: breast pain (96.9%), back pain (89.7%), dizziness (90.7%), chest tightness (92.8%), headache (83.5%), fatigue or weakness (78.4%), tingling sensation (78.4%), drowsiness (70.1%), numbness or paralysis (69.1%), and anxiety or nervousness (63.9%) (see Table 2). Despite these and other complaints, nothing was objectively found by the emergency medical team which evaluated these participants during and prior to the data collection periods. Exceptions were the presence of dry scar in the breasts of some of the study participants, bilateral breast lump in 2 (2.2%), axillary lymphadenopathy in 2 (2.2%), a breast ulcer in 1 (1.1%) women. The dry scar and breast ulcer happened to the study participants due to being cauterized by hot metallic-rode as a treatment by a traditional healer following the perceived unsatisfactory explanation and intervention by nearby health institutions.

**Table 2:** Commonly reported symptoms among the study participants

Symptoms	Number	Percent
Breast swelling	73	75.3
Breast pain	94	96.9
Back pain	87	89.7
Headache	81	83.5
Dizziness	88	90.7
Light headedness	39	40.2
Nausea	44	45.4
Abdominal cramps/pain	32	33.0
Cough	39	40.2
Fatigue/weakness	76	78.4
Drowsiness	68	70.1
Fainting/loss of consciousness	29	29.9
Sore or burning throat	34	35.1
Hyperventilation/difficulty of breathing	45	46.4
Chest tightness/pain	90	92.8
Watery or irritated eye	32	33.0
In ability to concentrate/trouble thinking	54	55.7
Vomiting	15	15.5
Diarrhea	9	9.3
Tingling sensation	76	78.4
Numbness/paralysis	67	69.1
Trouble with vision	31	32.0
Anxiety/nervousness	62	63.9
Skin rush/body itching	20	20.6
Tremor	26	26.6
Seizures like symptoms	6	6.2
Other (fever, sweating, loss of appetite, dyspepsia, neck stiffness, neck swelling, fear of death)	19	19.6

### Onset and duration of illness

The community members started to report symptoms from the beginning of 2012 up to the end of the data collection period, that is, June, 2015. Eighty (82.4%) of the women developed the symptoms in the years 2014 and 2015. Among these 50 (62.5%) developed the symptoms in the year 2015 while the rest developed the illness between 2012 and 2014. The duration of illness a woman suffered from the start of complaints ranged from 1 to 48 months, with a mean of 18.6 (SD ± 1.1) months.

### Information sources about the spread of cancer

Eighty-seven (89.7%) of the study participants said that they initially heard about the illness from indirect conversation or gossip. On the other hand, 42 (43.3%) of them reported that they had observed the illness directly from affected persons in addition to the gossip they had heard. Only 7 (7.2%) of the women said that they had heard the information either from health professionals or they couldn’t remember exactly from where they had heard the information first.

### Illness attribution and treatment sought by the victims

Fifty-one (52.6%) of the participants replied that they did not know the cause of the illness while 38 (39.2%) said it may be due to punishment by God. Twelve (12.4%) of the study participants reported that it may be due to the presence of toxic chemicals, polluted environment, cold air, using family planning injection or pills, influence of witchcrafts, and the curse of dead ancestor or evil spirits.

Three-fourth (75.3%) of the participants had sought treatment from modern health services and 62 (63.9%) had visited traditional healing services; while 42 (43.3%) had sought treatment from both modern and traditional healing services. Eight women (8.2%) had sought treatment from religious services; and 5 (5.1%) of the victims visited all the three treatment sites, namely traditional healers, religious healers and health institutions. Traditional healers either used hot metallic-rode to cauterize the affected area or ordered the individuals to apply herbal medicine or to drink herbal juice as treatment.

### Presence of mental illnesses and/or psychosocial stressors

Among the study participants, only three (3.1%) of them reported of having similar illness in the past and the same number of respondents admitted history of mental illness. Seventy-six (78.4%) of the study participants reported having had psychosocial stressors prior to the onset of the illness. Among them, 35 (46.1%) of them reported having psychosocial stressor just 1 month prior to the onset of the illness while 41 (53.9%) of them reported having one form of psychosocial stressor within 1 year prior to the onset of the illness. Eighteen (18.4%) of them had chronic psychosocial stressor. A death of close family or intimate friends (16.5%), personal health problems (16.5%), health problems within close family (15.5%), financial crisis (11.3%), quarreling with close family (10.3%), and either sexual or physical abuse (7.2%) were the commonly reported psychosocial stressor among study participants.

### Findings from qualitative responses

Two focus group discussions were conducted: one with affected women and the other with non-affected women. In each focus group discussion, seven women were involved. The ages of the participants ranged from 23 to 40 years. In addition to focus group discussions, four key informant interviews were undertaken. The key informants involved consisted of a health extension worker, a community leader, a Woreda health office head and a head of disease prevention and control officer. The information gathered during these discussions was grouped into the following three themes: Onset of the illness; understanding the nature of the problem and its possible transmission; and commonly reported symptoms and psychosocial impacts of the illness. Attempts were made to preserve participants’ phrases, experiences and meanings.

### Theme 1: onset of the illness

On the discussion made among participants about the onset of illness, a majority agreed that the onset of illness could be traced back to the year 2012 following the death of a 43 year old protestant, socially active and recognized woman. She was from Bussa Bassa Kebele. The woman was said to have had breast cancer and mastectomy was done in one of the government hospitals in the region. But after few months of the operation, the woman developed respiratory distress, loss of appetite, and experienced generalized body weakness. Finally, she died of cancer related complications in the second half of 2012. Before her death she had been showing the wound and the mastectomy site to woman closer to her. Though few women were complaining of having similar problems while the index case was alive, following her death, many women started complaining about breast swelling, breast and back pain. According to the majority of respondents, most of the affected groups were women closest to the index case by place of residence and blood.

### Theme 2: understanding the nature of the problem and its possible transmission

In both focus group discussions and key informant interviews, the theme did not specifically point at a single possible cause of attack and means of transmission. However, a majority of the participants said that their source of illness could be punishment by God caused by not obeying his commandments. Some of them believed they might have acquired the illness from contaminated water, pesticides, and polluted air. One mother said it could be secondary to using family planning injection or pills. As means of transmission, a majority believed it could be airborne, or transmitted through sitting with and drinking together with the affected person or else by taking toxic chemicals from contaminated water, food and/or air. A limited number of mothers said carrying heavy load, being overloaded with many duties at home and outside, having financial crisis and quarreling with close family could precipitate the illness. But they did not believe that these were the main contributors to the outbreak of the illness.

A majority of the respondents used to believe the cause of their suffering to be breast cancer; but through the repeated health education given since the beginning of 2015, their understanding has changed through time. They have heard from different health professionals that the ailments they are suffering from might not be cancer, but they did not find adequate explanations from health professionals for their worry. The findings also indicated that during KII’s also suggested the communities were suffering from a serious illness; but the root cause was still not clear to them. A 38 years old woman, expressing her belief and experience regarding the cause and means of transmission, said: “*I don’t know why we are suffering from this illness; it is the duty of God. People are suffering in mass, I don’t know its means of transmission; maybe we are getting it from contaminated water, food and air or it may be from the pesticides we use. I have visited different health institutions and traditional healers; the health professionals used to give some medication and the traditional healer cauterized the affected site using hot metallic rode, but both of them did not help me.*”

A 32 years old mother who expressed her deep concern also said: “*Our communities including me are suffering from a deadly ailment. Previously we were sure the cause of our suffering was breast cancer; but now because of the different information we have got we become confused about the possible cause of our suffering. We urge the government to tell us the exact cause of our suffering before we lose many lives.*”

### Theme 3: commonly reported symptoms and psychosocial impacts of the illness

The symptoms and signs revealed by FGDs and KIIs were almost similar to those reported by the afflicted participants. Symptoms centered on the breast. Manifestations were characterized by breast swelling, breast pain, back pain, squeezing sensation over the breast and chest. Some also complained of having numbness, tingling sensation and weakness in their upper extremity. A majority reported that the pain usually started on their back near their shoulder, and then migrated to their breast.

Because the illness was perceived to be contagious, this resulted in rejection by husband, family and neighbors. The stigma and discrimination were strong to the extent of not drinking and gathering together with the affected individuals. For many years the communities have had the tradition of drinking a local drink called “Cheka” by sharing the same drinking cup. But after the epidemic, nobody was willing to drink together in particular with the affected individuals. A majority of the participants feared risk of being divorced. There was mass terror among the community for fear of having the illness.

FGD with non-affected women showed that they were also worried about the situation. Some of them said that they will follow traditional treatment as their friends did because they believed there was no possible solution other than this. However, some of them reported that they will seek modern treatment if problems arise as they were getting health education at the time of rapid response. A 28 years old non-affected woman, expressing the degree of her fear about being affected by this illness, said: “*These days I and my neighbors have been on a great terror. Personally, I have been trying my best not to create contact with the affected person, but it is also difficult to distance oneself from the affected person, because we have lived together with them for long. In addition, some of them are our blood and family.*”

Women who participated in the FGDs said that the affected women had been reporting a constellation of signs and symptoms that looked like the presence of breast cancer like the woman who died with a complication of breast cancer. A 30 years old woman said: “*I don’t know what we can do. This disease is affecting our population; I am also one of the affected women. I heard the signs and symptoms of the disease from my colleagues but mine is not at that stage and I fear in the near future I will develop these signs and symptoms.*”

The above FGD findings were also supported by the KII findings. During Key informant interviews with individuals about the impact of disease in the public, one person said: “*Because of the community’s fear of being affected by the illness, schools were closed for few weeks.*”

On the other hand, the community’s limited medical facilities were overwhelmed. Moreover, primary health workers and Woreda health officials were baffled by the condition. The affected women were given different anti-pain and first line antibiotics, but a few days later, they started complaining about the symptoms again. The other problems reported by KIIs were the physical health impacts secondary to cauterization of the affected area with hot metallic-rode locally called “Astae”. During in-depth interviews with a traditional healer, Woreda health office head and health extension workers, they reported that a majority of the affected women usually visited traditional healers. The traditional healers, in addition to cauterizing the affected area, they were giving them herbal juice to drink and order them to apply herbs on the affected area.

Because of these interventions, some women developed massive infected wound over their breasts.

## Discussion

One hundred and twenty women were affected by the mass hysteria since 2012. From these, 104 women were involved in the study; 97 for quantitative study and 7 for qualitative study. The age of respondents who participated in the quantitative study ranged from 17 to 56 years, with a mean (SD) of 32.8 (8.7) years. The majority, 69 (71.1%) were from Bussa Bassa Kebele, the rest were from Bussa Killa Kebele. Ninety-two (94.8%) of them were followers of protestant religion. Like other previous studies [9, 13, 24], mass hysteria in this Woreda affected mainly females. In addition, like the previous studies [9, 13, 36, 37], the commonly reported symptoms were having some sort of physical pain, dizziness, chest tightness/pain, headache, fatigue, tingling sensation, breast swelling, drowsiness, and numbness or paralysis of the limbs. Significant numbers of women also reported having anxiety/nervousness, inability to concentrate/trouble thinking, hyperventilation/difficulty of breathing/, nausea, lightheadedness, cough, abdominal cramps, and fainting/loss of consciousness. The only difference from the majority of previous studies is the complaint of having breast cancer; this suggests to us that the other associated symptoms in this community follows illness anxiety; which can be defined as preoccupation of having or acquiring a serious illness without objectively having organic bases for the complaints [38]. Moreover, based on this finding we can say the community was suffering from chronic Mass Psychogenic Illness (MPI) for the following reasons: symptoms with no plausible organic basis, occurrence of symptoms in a segregated group, presence of chronic and acute anxiety, symptoms had been spread via sight and indirect conversation, preponderance of female patients and symptoms were similar with other studies [5, 39, 40]. The illness seems to have started at the beginning of 2012 following the death of a 43 year old protestant, socially active and publically recognized woman after being sick with breast cancer for long. The majority of the affected women were from Dubayisho Got where the index case had lived. The outbreak spread to the other adjacent areas as time passed. Though typically MPI affect the groups for limited periods [2, 5, 12], in this case it stayed for about three and half years. This might be because of the fragmented interventions and inadequate explanations given about causes and transmission of the presumed illness.

As to the causes of the illness, a majority were confused and perplexed. Before massive health education had been given, they used to believe they were suffering from breast cancer, but after health education they become confused as to what might be the cause. However, a majority said that their source of illness could be punishment by God for not obeying his commandments. Unlike our studies in many other studies this outbreak was attributed by the community to the work of evil spirits [10, 11, 37, 41] or the spirits of dead ancestors [23]. In Africa, many attribute MPI to supernatural causes such as witchcraft, Satanism or any element which makes up their cultural beliefs [42]. Like the western study [43], significant numbers of women in our study believed they might have acquired the illness either from contaminated water, pesticides, and polluted air. As to means of possible transmission, a majority of them believed it could be airborne from sitting and drinking together with affected person or else by taking toxic chemicals from contaminated water, food, and inhaled air. A limited number of mothers said that carrying heavy load, being overloaded with many duties at home and outside, having financial crisis and quarreling with close family members could precipitate the illness, but they do not believe that these are the main contributors for the outbreak of the illness. As a result, ordinary public activities were disrupted because of these beliefs. For example, many started to refuse to eat and drink with the affected women and schools were closed for few weeks. Searching for treatments and adequate explanations, many were moving here and there; from traditional to modern treatment sites. Unnecessary financial loss and iatrogenic illness were the major risks for these kinds of public behaviors. People working at all levels of the health sector in the Woreda were also confused and worried of the situation and lacked appropriate answers to the people complaining about the pain. They simply forwarded the peoples’ complaints to higher levels rather than teaching the community about the truths of cancer. This went on until an emergency response team from regional health bureau tried to clear community tension by doing massive psychoeducation.

Early identification and labeling of the condition is found helpful to control the spread of MPI. Among the crucial steps in managing MPI are: avoiding extensive investigation without letting down the victims’ complaints; being authoritative and firmly explaining that the outbreak is related to psychological conditions. Controlling the outbreak can be far-reaching if doctors continued extensive testing; since it strengthens the victims’ beliefs that their cause of suffering was either physiological or toxic agents. Moreover, telling the victims and their families that the condition has no relation to demonic possession or any organic illness could also bring huge reassurance and decrease the intense anxiety affecting the public [5, 44]. So in our population groups if early intervention and adequate explanation had been given from the outset of the illness, the outbreak could be easily contained within short period of time. It would have been also good if we include MPI as a training package in one of continuous professional development (CPD) going on in the country so that health professionals can be equipped with the necessary knowledge and skills to handle these kinds of outbreaks in the future. Awareness creation about these kinds of outbreaks to the community and people working in various media is also helpful. Unrealistic and exaggerated rumors and radio talk can be minimized by creating awareness to this group of the population. The emergency teams at different level in the health systems have to be as responsive as possible before this collective obsessional behavior reach out to other nearby communities and bring mass terror.

## Conclusion

Unrealistic and exaggerated rumors, non-organized and late intervention, inability to get adequate explanation about the causes and modes of transmission of the illness were the main contributing factors to the spread of the outbreak. Proper communication strategy can contain MPI within short period of time before they affect many individuals and bring about many psychosocial impacts on the community. Group anxiety could be reduced by providing proper psychoeducation timely and endowing convincing explanations.

## Abbreviations

MPI: mass psychogenic illness
SNNPR: Southern Nations, Nationalities, and Peoples’ Region
SPSS: statistical package for the social sciences
FGDs: focus group discussions
KIIs: key informant interviews
CPD: continuous professional development
